# Motor sequence learning data collected continuously for fifteen days of practice using a novel glove-based typing device

**DOI:** 10.1016/j.dib.2020.105234

**Published:** 2020-02-03

**Authors:** Dhanush Rachaveti, Varadhan SKM

**Affiliations:** Department of Applied Mechanics, Indian Institute of Technology Madras, Chennai, 600036 Tamil Nadu, India

**Keywords:** Motor sequence learning, Sequences, Typing task, Key press, And key release, Glove-based device, Finger thumb opposition task

## Abstract

The dataset presented in the article includes the timestamp of key press and key release data of individual participants during a novel finger thumb opposition typing task. The novel task involves touching different segments (phalanges) of fingers with the thumb to type a specific symbol on the computer screen. This task involves learning of set of sequences by typing or touching them using the finger thumb opposition movements is termed as motor sequence learning task or paradigm. The symbol set comprised of nine most frequently used symbols in English. From the nine symbols, a set of 281 meaningful five lettered words (sequences) were formed. These sequences were presented to the participants in a game-like interface. Once a specific symbol was pressed and released the time stamp was registered in the computer as key (symbol) press and key release information. The dataset consists of three columns, first column shows the pressed key, second column the registered timestamp and final column shows the symbol activity with respect to the first symbol in terms of milliseconds. Key press information is followed by key release information. This is represented in the dataset as “LCONTROL” in the first column of the data. Changes of this key press and key release information over the course of practice can be used to understand change in performance of this novel tying task.

**Table:** Specification table:

Subject	Behavioural Neuroscience
Specific subject area	Motor sequence learning
Type of data	Figures, tables and Key press and key release data (timestamps).
How data were acquired	The data was acquired using a glove-based typing device developed in our lab. Customized software was developed using LabVIEW to collect the data from glove-based typing device at 1000Hz
Data format	Raw data,.txt format
Parameters (experimental factors) for data collection	The data was segregated as different practice sessions (15 days) and different blocks of practice (12) within each practice session. Hence practice session and blocks on a given day can be considered as two experimental factors.
Description of data collection (experimental features)	Each practice session consisted of 30 minutes of data collection (2 min x 12 blocks; 30 seconds rest between blocks) per day. There were fifteen such sessions on fifteen consecutive days (including weekends/holidays). Participants performed the gloved based typing task in the lab every day. This experiment was performed to explore how learning evolves in a novel glove-based typing task using nine symbols and 281 sequence set.
Data source location	Chennai, TamilNadu, India
Data accessibility	https://doi.org/10.17632/jhwz4gzcsb.2

**Table:** 

**Value of the data** • The data can be used to understand how learning of a novel task evolves or changes with substantial practice over a period of time. • The data is valuable as the participants physically came to the lab and performed the task continuously for fifteen days unlike the similar learning experiments that involve home training sessions. • The dataset presented in this article will help people who work extensively in the field of cognition for understanding the cognitive aspects of learning, such as memory consolidation, representations, and sleep patterns [[1], [2], [3], [4]]. • The present data set includes nine symbols and 281 sequences. Hence such large dataset can be used to understand context-specific learning similar to the domains of American sign language, handwriting, piano playing, conventional typing [[5], [6], [7], [8]]. • New experiments in the field can be designed by changing the number of symbols and sequences to address a specific phenomenon of interest. • The performance measures determined from the current data set will enable future experimenters to find when the performance saturates or in other words when to stop such learning experiments.

## 1 Data

**Fig. 1:**
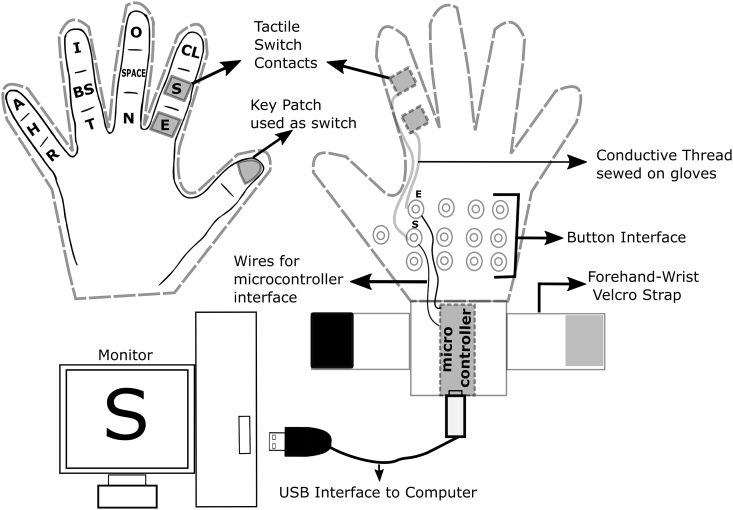
**Block diagram showing the functioning of the glove based typing device.** Participants wore gloves (showed with dotted lines) such that the tactile switches on the glove faced towards the participants. The hand is represented with bold lines. Key patches (showed as square around the alphabets) were sewed on the dorsal side of each finger segment on the gloves. Conductive threads, sewed on the glove were used to connect the key patches to a button connector, which was used to interface with the microcontroller. When an symbol patch was touched with thumb, a custom-written code in the microcontroller converted the touch into text. This was shown on the computer monitor.

**Fig. 2:**
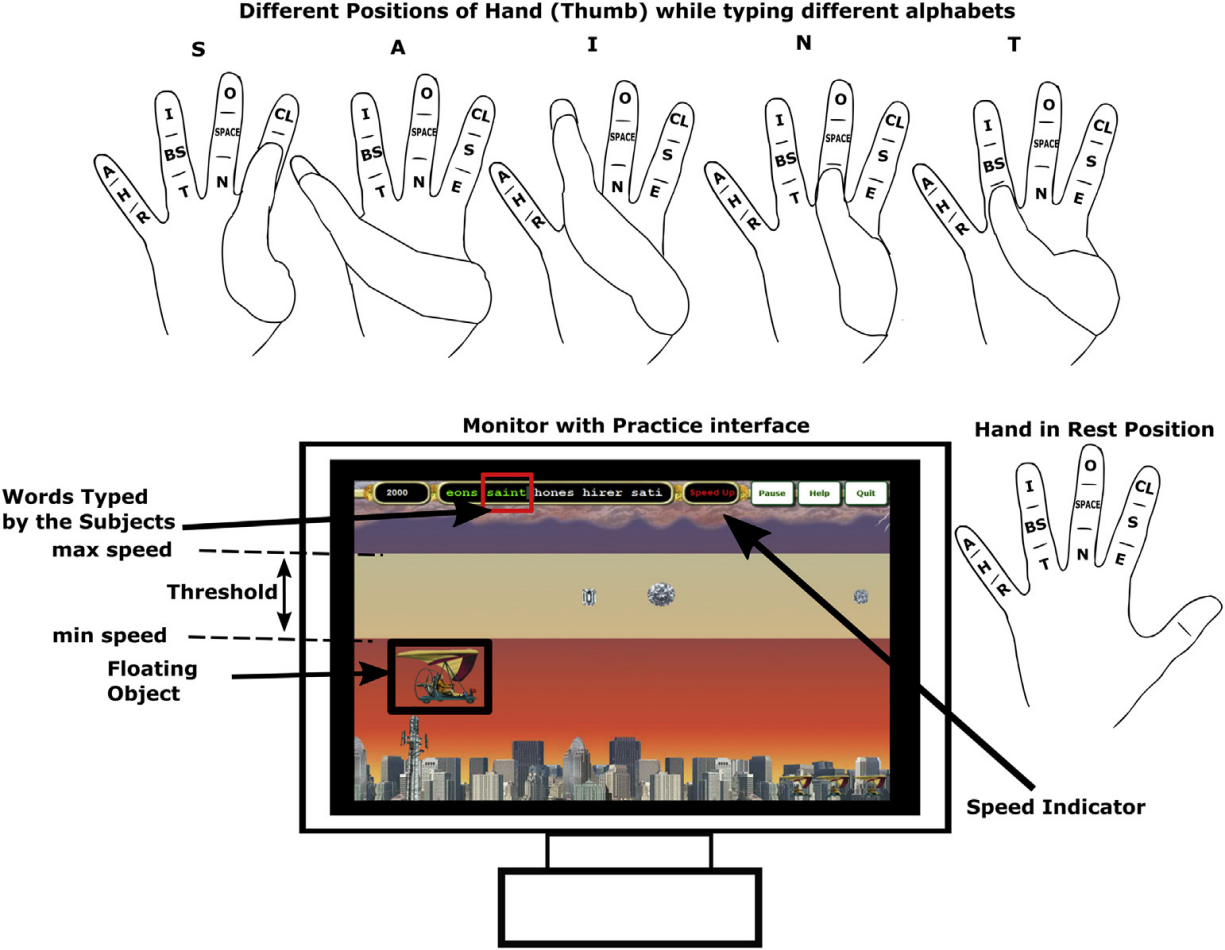
**Practice interface used in the experiment, a.** The sequence of finger opposition movements performed by participants to type the word “SAINT”. Practice interface (Game) used in the experiment shows movement words from right to left as participants typed the words. The word “SAINT” shown on the game was typed correctly and hence the word was highlighted in green color. The objective of the game was to type the words as fast and accurately as possible so that the Glider moves from left to right towards the destination. The object will lose altitude and crash with low speed and accuracy (high errors). SP, CL, BS denotes “SPACE”, “Caps Lock”, “Back Space”. Participants begin the experiment with hand in the rest position. b. Each symbol when typed two information were logged one is Key Press (KP) and another is Key Release (KR). Using this two information, Dwell time (DT) and movement time (MT) were calculated for each symbol. Completion time (CT) is calculated for a word.

The dataset presented in the article was collected using a glove based typing device as shown in Fig. 1. The right handed participants were recruited to use the glove based typing device to type the words shown in a game like interface as seen in Fig. 2. The words shown to the participants are tabulated in Table 2. The data collected is organized into folders and subfolders for easy accessibility and reusability by fellow researchers in the field. The folder hierarchy is as follows, root folder signifies the participant ID followed by subfolders indicating different practice sessions (Day 1 to Day 15). The practice session subfolder consists of twelve text files for each of the twelve blocks performed in a given practice session. These text files consist of three columns as shown in Table 1. First and second columns indicates the key activity and its corresponding time stamp of the activity. While, the third column indicates the key press and release timestamp information of all the letters typed in a given block with respect to the first key press. Hence, for the first key press, the third column value in any block would be the zeroth instance indicated as zero in these files. The information of all the participants recruited for the experiment can be seen in Table 3.

**Table 1:** **Data representation in data file:** The column shows the keypress and release activity of a specific letter (in this case “S” and “T”). Column 2 represents the time stamp of the key press and release activities. The third column indicates key press and release activities in terms of seconds.

Column 1	Column 2	Column 3
Letters typed	Timestamp	Time in seconds with respect to the first letter typed.
S (Key press)	DD-MMYYYYhhmmss.yy	x.xx (seconds)
LCONTROL (Key release of “S”)	DD-MMYYYYhhmmss.yy	x.xx (seconds)
T (Key press)	DD-MMYYYYhhmmss.yy	x.xx (seconds)
LCONTROL (Key release of “T”)	DD-MMYYYYhhmmss.yy	x.xx (seconds)

**Table 2:** **List of words shown to participants in every block:** Words of five letter length shown to the participants from blocks one to twelve (B1 to B12). Words could repeat within a block but not between blocks, and words on a given block remained same across all days. All blocks had approximately 23 number of words except the last block of practice, which had five sequences extra than other eleven blocks (28 sequences).

B1	B2	B3	B4	B5	B6	B7	B8	B9	B10	B11	B12
aeons	sitin	atone	tonne	short	tires	senna	toner	shins	rinse	norse	heron
ashen	trots	noons	sorts	sties	rants	noses	shore	tress	tiara	hints	arson
easer	rises	shone	shoer	eater	risen	snits	shies	raise	roars	shoes	saith
hirer	stern	honor	sarin	saris	henna	snore	tarsi	inert	toast	tenth	areas
hones	arose	tatar	state	antra	sites	stare	tithe	siren	ashes	rites	arise
rarer	rears	tones	stash	anise	hares	iotas	horse	thens	trees	teeth	tooth
reins	snort	arias	rents	trash	satan	tarts	nears	reset	roost	shier	honer
roans	seers	oases	titan	sates	stent	sheet	enter	sires	there	their	atria
saint	nines	nests	irate	trent	oasis	asset	heirs	sheen	oaths	tenet	eosin
satin	nares	hater	ratio	tarot	tents	heart	thats	north	taste	totes	rains
setae	store	start	ethos	sines	neons	stats	asses	tense	hoser	inset	torte
sitar	trite	theta	tines	those	tetra	taros	nitro	shire	hairs	eaten	onset
snots	heros	steno	toter	seine	soots	share	terse	tints	stain	sense	eases
sonar	hosts	rests	notes	earns	ester	naira	hoses	sorer	trier	thine	shear
stars	thorn	ninth	rosin	hoist	ether	shahs	airer	trios	sores	onion	tenor
stein	seres	otter	rhino	other	rares	saner	orate	reran	three	tares	roist
taint	senor	sheer	stair	torso	stone	train	arena	resit	tears	hires	renin
teats	sears	eerie	heath	teens	inter	heist	tinea	inner	astir	sneer	shoot
tenon	irons	erase	hears	aster	roses	riots	hoots	snare	heres	harsh	thins
terns	retie	hosta	shine	shish	soars	rater	shots	roots	haste	snoot	asher
tests	heats	these	noise	trait	horns	resin	tiers	tatoo	aerie	shorn	hates
throe	anion	retro	shirt	stirs	aorta	tarns	torts	stint	tries	ortho	sanes
tsars	rates	serer	roast	toots	resat	steer	riser	seats	inane	treat	torah
											error
											rotor
											earth
											tease
											noose

**Table 3:** **Participant information:** The participants recruited for the experiment were six males and two females. Only right-handed (RH) participants were recruited with no prior history of finger or hand fracture. The experiment was conducted for participants based on their circadian rhythm, the inventory showed that they were “Morning”/“Evening” people, then the experiment was conducted during forenoon session. Participants were instructed to come maintain same timing for next practice session till the last session. They were also instructed to have 8 hrs sleep during the experiment days.

S.No	Participant id	G	Age	Weight (Kg)	Height (cm)	Handed condition	Hand/finger fracture	Continuous practice sessions	Sleep hrs	Circadian rhythm
1	S1	M	29	65	179	RH	No	15 days	8 hrs	Morning
2	S2	M	24	68	171	RH	No	15 days	8 hrs	Evening
3	S3	M	28	83	174	RH	No	15 days	8 hrs	Evening
4	S4	M	29	74	173	RH	No	15 days	8 hrs	Morning
5	S5	M	25	70	169	RH	No	15 days	8 hrs	Evening
6	S6	M	29	83	167	RH	No	15 days	8 hrs	Evening
7	S7	F	27	54	153	RH	No	15 days	8 hrs	Morning
8	S8	F	26	66	155	RH	No	15 days	8 hrs	Morning

## 2 Experimental design, materials, and methods

### 2.1 Statement of ethics and recruitment of participants

Eight healthy, right-hand dominant participants (6 males & 2 females, mean ± sd: Age: 26.8 ± 2.6 yrs., Height: 163.5 ± 6.0 cm, Weight: 66.8 ± 10.5 kg) volunteered for the experiment. Participants had no history of any neuro-motor disorder or trauma to the hand or fingers and were naïve to the purpose of the experiment. Handedness was determined using Edinburgh handedness inventory, and all participants who had a handedness score above 90% (score of 90 and above indicates that participants were right hand dominant) were recruited [9]. Participants were tested with a self-assessment human circadian rhythm inventory to determine whether they were morning type or evening type people according to their circadian rhythm [10]. This inventory was shared with the recruited participants before the commencement of the practice schedule. We scheduled the experiments appropriately in mornings or evenings based on this inventory. All volunteers for the experiment were right-handed. The participants provided written informed consent before participating in the experiment. All experimental procedures were approved by institutional ethics committee of the Indian Institute of Technology Madras (Approval number: **IEC/2016/02/VSK-12/22**).

### 2.2 Experimental setup

Our experimental system was a glove-based typing device. This system consisted of a glove with conductive key patches placed approximately at the center of each segment in index, middle, ring, and little fingers (3 segments * 4 fingers = 12 keys) and one at the distal thumb (tip). Among these 13 keys, the one on the thumb was used as a switch, while the 12 on the other fingers were assigned with nine specific symbols, space, backspace, and caps lock. These keys were connected to a microcontroller (Teensy 2.0++) using conductive thread, metallic buttons, and cables. Fig. 1 illustrates the experimental setup and the keymap used. In order to type a particular letter, participants had to touch the corresponding key patch on a finger with the thumb, which then closed a specific electrical circuit. A customized program in the microcontroller detected this event, and the program then sent the ASCII code of that specific key to the computer through a USB port. For example, when the participant touched middle phalanx of the index finger, symbol ‘S’ was typed on the computer screen as shown in Fig. 1. These gloves were custom-made to suit the hand dimensions for each participant. The text from the glove was processed by a customized LabVIEW based program at 1000 Hz.

### 2.3 Motor sequence learning task

Participants wore the glove and were instructed to make opposition movement with the thumb as seen in Fig. 1, to the finger phalanx to type-specific symbols. For example, when the participant touched middle phalanx of the index finger, symbol ‘S’ was typed on the computer screen as shown in Fig. 1. Hence, the task involved learning of set of training sequences by typing them using their finger thumb opposition movements. This task is known as motor sequence learning task or paradigm.

### 2.4 Training sequences

Sequences used in the experiment were 5-letter words picked from a custom dictionary (Please see Table 2 for sequences used in the experiment). This dictionary comprised 281 sequences, each made using five of the nine most frequently used symbols (e, s, o, n, i, t, a, r, h). These symbols were mapped to keys on the glove to form a keymap, as shown in Fig. 1 (placed on the gloves). For all participants, the same keymap was used on all days and blocks. First eleven blocks comprised of 23 sequences, while the twelfth block had five more sequences than other blocks of the practice session. Hence, the total number of sequences were 23 × 11 = 253 + 28 (12th block) = 281 sequences. These 281 words (“sequences”) were the only words that had a meaning in the English language. These 281 sequences were equally distributed as 23 sequences across 11 blocks and 28 sequences for the 12th block.

### 2.5 Game interface

The sequences typed by participants were displayed in a game interface (Diamond glider game in Typing Instructor® Platinum 21, Individual software, CA, USA), as shown in Fig. 2. The objective of the game was to type sequences quickly and accurately to move a glider from the starting point to destination without crashing. The glider moved towards the destination as the participants typed. Sequences to be typed appeared on the right and moved left on the screen as the participant typed them. If a correct letter was typed, that letter was highlighted in green, and the cursor moved to next letter. If a wrong letter was typed, that letter was highlighted in red, and the cursor stayed on the same letter until the correct letter was typed. An audible beep tone was played when an error occurred. In addition to the sequences, participants had to type SPACE key in between sequences.

The game interface had an option to prescribe minimum typing speed for a block. This option was used by the experimenter to set minimum typing speed before each block. Also, the game algorithm also set a maximum speed, which was five words per minute (WPM) above this experimenter-chosen minimum speed. The participants were instructed to type as many sequences as possible correctly so that the glider stayed above the minimum speed If the errors made were greater than letters typed per minute or if speed was slow (as decided by the game algorithm), the glider crashed and the game was over. Likewise, if they typed greater than maximum speed, the glider could hit the ceiling and crash (although this never happened in our experiments). When the participant typed below minimum speed the speed indicator displayed “speed up” and when the participant was about to hit the ceiling (close to the maximum speed) the indicator displayed “speed down” as shown in Fig. 2.

For all participants, the minimum speed was set to 5 WPM (words per minute) in the first block on Day 1 of practice. If the experimenter observed that the participant performed well at the prescribed minimum speed and was typing close to the maximum speed, the minimum speed was incremented by 5 WPM for the next block.

### 2.6 Software (LabVIEW) control

A customized code in LabVIEW was used to register the key pressed and released while playing the game using glove-based typing device. The LabVIEW starts to monitor the key press and key release activity before the start of the game. Once the participants start to type, the game begins, but the LabVIEW monitors the key press and release activities of the participants even before the beginning of the game. Hence the third column in the data always have a few seconds data more than the duration of each block (120 seconds). The keypress and key release information for a period of 120 seconds from the first key press in a given block were considered for calculation of performance measures such as completion time, dwell time, movement time and errors.

### 2.7 Experimental protocol

Participants practiced the experimental task for 15 consecutive days, and data were collected on all days of practice. Each day/session was divided into 12 blocks of 2 mins, each with 30 seconds interval between each block. Sequences could repeat within a block but not between blocks, and sequences on a given block remained same across all days (i.e. Block “m” was composed of same set of sequences on all days but the order of sequence presentation may change between days; block n always had a set of sequences different from block m, when m≠n). All blocks had approximately 23 number of sequences (Table 1).

### 2.8 Data analysis

The present dataset can be further analyzed by computing the performance measures such as accuracy and speed attributes of the typing task. The sequences presented to the participants on each block are shown in Table 2. From these presented sequences and typed sequences errors can be determined as accuracy attributes. Based on the time of pressing and releasing a key speed attributes can be computed.

**Accuracy attributes:**

**Errors (ER):**

Errors can be calculated as the total number of letters typed wrongly in a given block and averaged across all blocks for a specific day of practice.

**Speed attributes:**

Timing parameters like key press and release were collected during all days of practice and can be analyzed further using metrics defined below.

**Key Press time (KP):**

Key Press time is defined as that time when the thumb makes contact with the tactile key patches on the finger phalanges.

**Key Release time (KR):**

Key Release time is defined as that time when the thumb is removed from the contact with the tactile key patches on the finger phalanges.

From the KP, KR information, the following parameters can be computed. KP and KR can be seen as time stamp information in the second column in the dataset. The third column in the dataset is the KP and KR information of the other letters typed relative to the first letter typed. Below performance measures can be determined using the KP and KR information from the third column of the data set.

**Completion Time (CT):**

Completion time is defined as the time taken to complete a sequence, which can be computed as the difference between key press times of last letter (fifth letter) and first letter as shown in Fig. 2b.

**Dwell Time (DT):**

Dwell time is defined as the time spent on each key (letter) in a sequence, which can be computed as the difference between key release time and key press time for a specific symbol (letter) as shown in Fig. 2b.

**Movement Time (MT):**

Movement time is defined as the time taken to reach/press a particular symbol (letter) after the release of the previously typed symbol (letter), which can be computed as the difference between key press time of the specific symbol (letter) and the key release time of the previously typed symbol as shown in Fig. 2b.

### 2.9 Data identification

The data file was organized within the root folder named with the participant ID followed by subfolder named with every practice session number (Day 1 to Day 15). Each practice session consists of twelve data files for each block practiced in a given practice session. The data file data_block_(block number).txt consists of three columns-column- 1, column- 2, column- 3. Table 1 illustrates three columns in the data file. In column- 1 letter “S” indicates that the specific letter was pressed and “LCONTROL” immediately in the next row indicates the release activity of the letter “S”. The corresponding time stamp and duration of key press and release information in terms of seconds was stored in column- 2 and column- 3. The timestamp convention is as follows date-month-year-hours-minutes-seconds-milliseconds. Table 3 shows the information on the participants recruited for the experiment.

## References

[1] Karni A. (1996). The acquisition of perceptual and motor skills: a memory system in the adult human cortex. Cognit. Brain Res..

[2] Kami A., Meyer G., Jezzard P., Adams M.M., Turner R., Ungerleider L.G. (1995). Functional MRI evidence for adult motor cortex plasticity during motor skill learning. Nature.

[3] Rozanov S., Keren O., Karni A. (2010). The specificity of memory for a highly trained finger movement sequence: change the ending, change all. Brain Res..

[4] Doyon J., Korman M., Morin A., Dostie V., Tahar A.H., Benali H., Karni A., Ungerleider L.G. (2009). J. Carrier, Contribution of night and day sleep vs. simple passage of time to the consolidation of motor sequence and visuomotor adaptation learning. Exp. Brain Res..

[5] Jerde T.E., Soechting J.F., Flanders M. (2003). Coarticulation in fluent fingerspelling. J. Neurosci..

[6] Wright C.E. (1993). Evaluating the special role of time in the control of handwriting. Acta Psychol..

[7] Winges S.A., Furuya S., Faber N.J., Flanders M. (2013). Patterns of muscle activity for digital coarticulation. J. Neurophysiol..

[8] Flanders M., Soechting J.F. (1992). Kinematics of typing: parallel control of the two hands. J. Neurophysiol..

[9] Oldfield R.C. (1971). The assessment and analysis of handedness: the Edinburgh inventory. Neuropsychologia.

[10] Horne J.A., Ostberg O. (1976). A self-assessment questionnaire to determine morningness-eveningness in human circadian rhythms. Int. J. Chronobiol..

